# Efficacy and safety of moxibustion for patients with functional constipation: A protocol for systematic review and meta analysis

**DOI:** 10.1097/MD.0000000000020910

**Published:** 2020-07-10

**Authors:** Ying Chen, Mingmin Xu, Tinghui Hou, Lu Wang, Xiumei Feng, Ying Li

**Affiliations:** School of Acupuncture and Moxibustion and Tuina, Chengdu University of Traditional Chinese Medicine. No. 37 Shierqiao Road, Jinniu District, Chengdu, Sichuan, China.

**Keywords:** functional constipation, moxibustion, protocol, systematic review

## Abstract

**Introduction:**

The objective of this review is to assess the efficacy and safety of moxibustion for treating patients with functional constipation (FC).

**Methods and analysis:**

We will electronically search the following databases: OVID MEDLINE, EMBASE, PubMed, Web of Science, the Cochrane Central Register of Controlled Trials, Cochrane library, CINAHL, AMED, China Network Knowledge Infrastructure, Wan-fang Database, China Biomedical Literature Database, and other resources from inception to October 2019, without any language restrictions. Randomised-controlled trials will be included. The primary outcome is the improvement in mean complete spontaneous bowel movements and stool form (utilize the Bristol Stool Form Scale [BSFS]). Secondary outcomes involve the degree of difficulty in defecation, proportion of responders, mean transit time, health-related quality of life, and adverse events rate. The methodological quality will be assessed using the Cochrane risk of bias tool.

**Results:**

This work will summarize clinical evidence to assess the effectiveness and safety of moxibustion treatment for FC patients.

**Conclusion:**

This systematic review and meta-analysis will provide current evidence of the efficacy and safety of moxibustion treating FC.

**Systematic review registration:**

PROSPERO, CRD42020157955.

## 1 Introduction

Functional constipation (FC) is characterized clinically by spontaneous defecation less than 3 times per week, incomplete defecation feeling, dry stool, etc, according to the Rome IV criteria.^[1]^ It is a prevalent clinical disease without any specific physiological changes.^[2]^ Several epidemiological studies have reported that the prevalence of constipation in the general population is ∼16%, although it can range from 2% to 27%, depending on the definition used and population studied.^[3–6]^ Constipation symptoms significantly reduce patients’ quality of life, mentally, and physically.^[7,8]^ A study showed that 89% of constipation patients still reported constipation during follow-up period of more than 12 months.^[3]^ Additionally, it is reported that constipation is related to a higher possibility of digestive system symptoms such as abdominal pain, gas, and nausea.^[2,9,10]^ Considering that FC makes a significant adverse impact on patients’ quality of life and economic costs,^[11,12]^ it should be considered as a major public health problem.

There are 3 broad categories of therapies are used to manage constipation symptoms for patients with FC, such as surgical, pharmacological, and nonpharmacological.^[13,14]^ Surgical therapy has strict indications and is performed only in severe cases, constipation surgery is not common.^[15,16]^ It has been reported that pharmacological therapies such as osmotic and laxatives, and selective 5-hydroxytryptamine receptor 4 (5-HT4) agonists are reported with definite efficacy.^[17–19]^ But constipation tends to recur when the usage of these drugs stops, and more adverse events happen in patients receiving these treatments.^[19,20]^ Non-pharmacological therapies are popular among FC patients, such as increasing water intake,^[21]^ adding fiber to the diet,^[22]^ and exercising,^[23]^ but the effect is weak.^[9]^ Thus, increasing patients with FC usually seek additional effective, safe and non-toxic alternative therapies.^[24]^

Acupuncture, an important component of traditional Chinese medicine (TCM), has been used for thousands of years to treat gastrointestinal problems including diarrhea and constipation.^[25,26]^ Moxibustion also plays an important role in the prevention and treatment of numerous diseases and often accompanies acupuncture treatment.^[27]^ Moxibustion is a natural therapy suitable for some chronic and severe diseases, and works by stimulating acupoints with heat energy from ignited moxa.^[28]^ TCM theory holds that moxibustion treatment promotes qi stimulation and resolves qi stagnation at an acupoint.^[29]^ Systematic reviews have reported the effectiveness of moxibustion in several diseases, including insomnia, hypertension, irritable bowel syndrome, and constipation.^[30–33]^

However, a systematic review that focuses especially on the moxibustion treatment for FC has not been published so far. This protocol will summarize the current evidences and conduct a systematic review and meta-analysis to appraise the efficacy and safety of moxibustion for patients with FC.

## 2 Methods and analysis

### 2.1 Design and registration of the review

Our protocol for this systematic review has been registered on PROSPERO (registration number is CRD42020157955) and the protocol is designed strictly follow the Preferred Reporting Items for Systematic Reviews and Meta-Analyses meta-analysis protocols (PRISMA-P) statement guidelines.^[34]^ The PRISMA-P Guidelines and the Cochrane Handbook will be used for the studies we evaluate for inclusion. In addition, bias risk analysis and heterogeneity analysis will also be used in this protocol. Subgroup analysis and sensitivity analysis will be further carried out when necessary.

### 2.2 Inclusion criteria

#### 2.2.1 Type of study

Only randomised controlled trials (RCTs) will be included in this protocol. However, studies that used incorrect randomization methods (such as flipping a coin) would not be included. Any other type of literature will be excluded, including moxibustion literature as a non-primary intervention, retrospective research literature, repeated publications, conference abstracts, literature that cannot extract data, case reports, and bibliometric studies.

#### 2.2.2 Types of participants

Patients diagnosed with FC according to the Rome II, III, or IV criteria will be included in this review. Trials studies of FC due to specific pathological cause, such as underlying structural or metabolic diseases will be excluded. There will be no gender, age, race, nationality, education status, and economic status.

#### 2.2.3 Types of interventions

We plan to include trials involved any type of moxibustion, such as direct moxibustion, indirect moxibustion (such as cake-separated moxibustion, ginger-separated moxibustion), moxa burner moxibustion, heat-sensitive moxibustion, natural moxibustion, and crude drug moxibustion, regardless of the treatment material, frequency, duration, and method. Trials in which moxibustion is not used as a major therapy will be excluded. Moxibustion therapy will be compared with non-moxibustion therapy, placebo moxibustion control (such as moxa stick not ignited), and no treatment.

#### 2.2.4 Types of outcomes

The primary outcomes of this review will be the improvement in mean complete spontaneous bowel movements and stool form (utilize the Bristol Stool Form Scale [BSFS]).^[2]^

The secondary outcomes will be: the degree of difficulty in defecation, proportion of responders, mean transit time, health-related quality of life, and adverse events rate. The degree of difficulty in defecation include defecation interval time, lumpy, or hard stools, soiling and blood-stained stool, sensation of anorectal obstruction/blockage, difficult defecation, and encopresis. The proportion of responders is defined as the number of responders divided by the total number of participants in each group. Transit time is the time from the first perception of wanting to defecate to the finish of defecation, and the mean transit time will be calculated. The health-related quality of life will be measured by the Medical Outcomes Study 36-Item Short Form Health Survey (SF-36), which is normally used by constipation studies. And we will calculate the proportion of adverse event rate.

### 2.3 Data sources and search methods

#### 2.3.1 Electronic searches

We will electronically search the following databases: OVID MEDLINE, EMBASE, PubMed, Web of Science, the Cochrane Central Register of Controlled Trials, Cochrane library, CINAHL, AMED, China Network Knowledge Infrastructure, Wan-fang Database, China Biomedical Literature Database from inception to October 2019, without any language restrictions. The search strategy will be developed after a discussion among reviewers, according to the guidance of the Cochrane handbook.^[35]^ The following search terms will be used: FC, chronic constipation, idiopathic constipation, slow transit constipation, constipation, functional gastrointestinal disorder, functional defecatory disorder; moxibustion, moxa leaf, moxa velvet, moxa stick, moxa cone, moxibustion box, cake-separated moxibustion, ginger-separated moxibustion, dragon moxibustion (Du meridian moxibustion), heat-sensitive moxibustion, suspension moxibustion. The search strategy for PubMed is shown in Table 1. This search strategy will be slightly modified and used in several other databases.

**Table 1:**
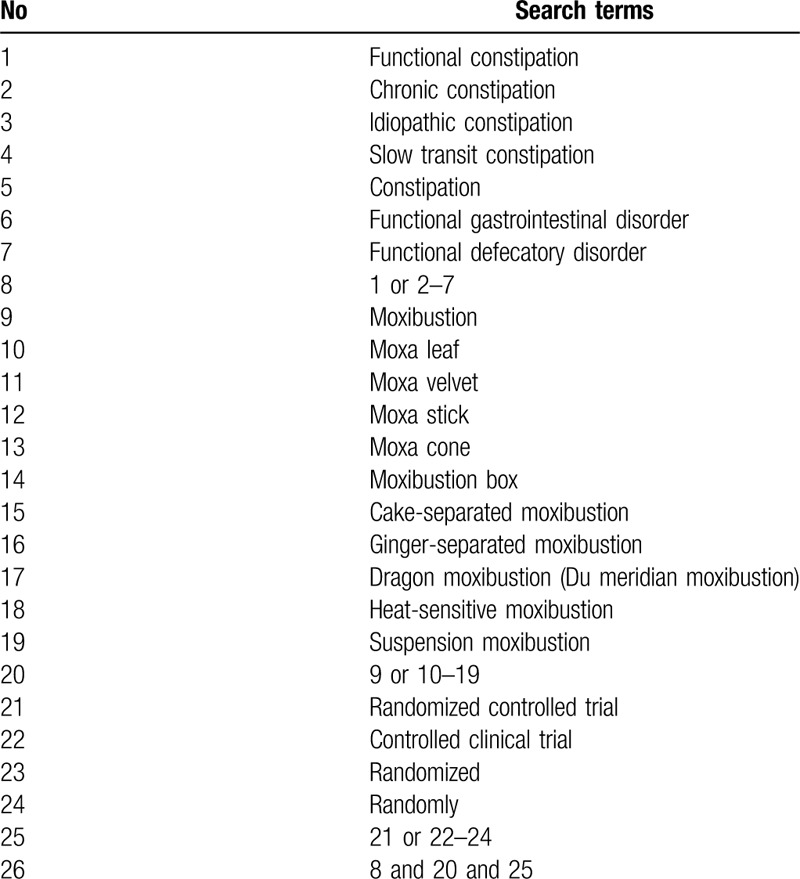
The search strategy used in Pubmed.

#### 2.3.2 Searching other resources

PROSPERO, the International Clinical Trials Registry Platform, and ClinicalTrials.gov will also be searched to identify systematic reviews or ongoing/completed clinical trials. We will conduct a hand search of relevant journals and their conference proceeding. Theses and bibliographic references of included trials will also be reviewed.

### 2.4 Data collection and management

**Figure 1:**
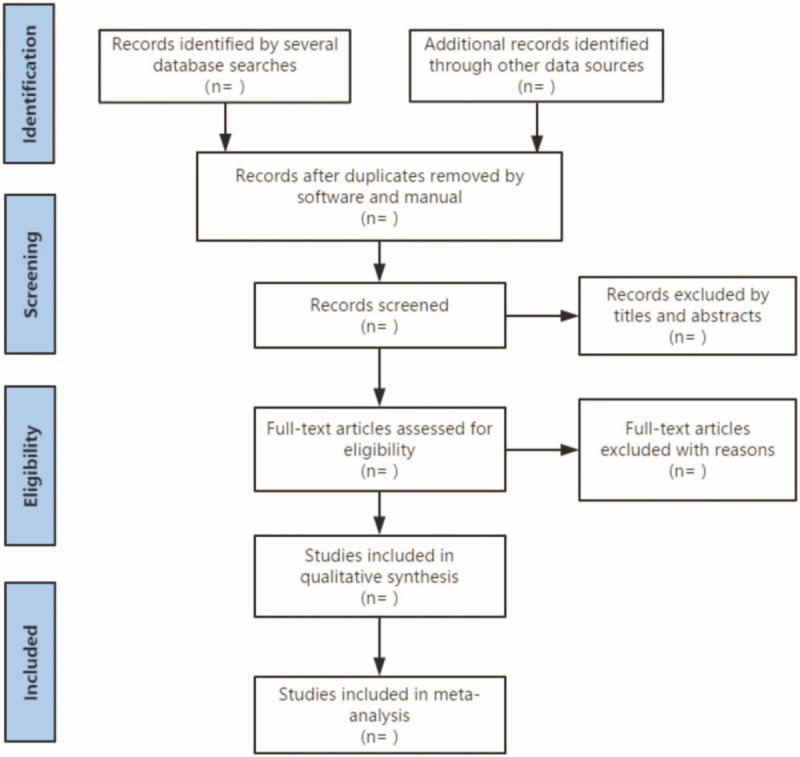
Flow diagram of study selection. This picture reflects the steps of research selection, and explains the process of literature screening in detail.

Prior to the literature search, a procedure for screening hosted by YL, will be discussing among all the reviewers, to ensure consistency in the evaluation of this study. After electronic searches, the result will be imputed to Noteexpress software Version 2.6.1 (Aegean Sea software company Beijing, China) in the same text format, and repeated research will be eliminated by software. Two reviewers (YC and MMX) will independently complete the screening of documents, then cross-check to determine the final inclusion of documents. In the first stage, all documents after software review will be screened for title, summary, and keywords to meet the selection criteria. In the second stage, we will evaluate the full text of the remaining studies and determine whether it meets the systematic review's criteria. Once any disagreement occurs, it will be settled by discussion, or a decision will be adjudicated by a third reviewer (YL). Details of entire study selection procedure are summarized in Figure 1.

### 2.5 Data extraction and analysis

Before data extraction, a standard data extraction form (Excel) containing specified outcomes will be created according to the inclusion. Two researchers (THH and LW) will then extract data and analysis independently. The data will include the first author, country, year of publication, number of participants, patient characteristics, study duration, funding source, interventions, outcomes indicators, main conclusions, conflicts of interest, recurrence rate, acupoint selection and adverse events. In this process, any disagreements will be resolved by discussion between the 2 reviewers, if necessary, final determination from a third reviewer (XMF) will be sought.

### 2.6 Assessment of risk of bias in the included studies

Two reviewers (HTH and LW) will use the “Risk of bias” tool in Cochrane Manual V.5.1.0 to evaluate the bias risk of the included trials.^[35]^ The evaluation includes random sequence generation, allocation sequence concealment, blinding, incomplete data reporting, selective result reporting, and other bias sources. If the risk of bias is high in the literature, we will try to explain and discuss the causes of bias.

### 2.7 Assessment of heterogeneity

We will test the heterogeneity of data by calculating the value of *I*^2^ statistics and *χ*^2^ test. When *P* > .1, *I*^2^ < 50%, it is considered that there is no great heterogeneity in the study. When *P* < .1, *I*^2^ > 50%, it is considered that the study has significant statistical heterogeneity. At this very moment, the subgroup stratification analysis will be further carried out to explore the possible sources of heterogeneity.

### 2.8 Assessment of reporting bias

If our review has a sufficient number of included trials that are available in the meta-analysis, a funnel plot and statistic test will be generated to analyze the potential reporting bias as well as small study effects.

### 2.9 Data synthesis

For continuous data, we will use mean difference (MD) or standard MD (SMD) to measure the therapeutic effect of 95% CIs. If significant heterogeneity is found, we will use the random-effects model instead. For dichotomous data, we will denote the outcomes as relative risks (RRs) with 95% CIs. If the *I*^2^ test is <50%, the fixed-effects model will be used for data synthesis. If the *I*^2^ test is between 50% and 75%, the random-effects model will be conducted for data synthesis. If the *I*^2^ test is higher than 75%, we will investigate possible reasons from both clinical and methodological perspectives, and provide a descriptive analysis or conduct subgroup analysis.

### 2.10 Subgroup analysis

In the case of high heterogeneity, subgroup analysis will be done to identify the sources of heterogeneity. We will conduct subgroup analysis according to different combinations of moxibustion and other combined therapies, different course time or different outcome indicators.

### 2.11 Sensitivity analysis

When there are adequate studies, sensitivity analysis will be adopted for primary outcomes to explore the robustness of conclusions if feasible, and assess the impact of method quality, sample size, and missing data. The meta-analysis will be conducted again after excluding the lower quality research. The results will be compared and discussed according to the results.

### 2.12 Grading the quality of evidence

The quality of evidence and confidence for the main outcomes of systematic reviews included in this review, will be evaluated by using the Grading of Recommendations Assessment, Development and Evaluation (GRADE) guidelines.^[36]^ The quality of evidence will be adjudicated into four levels: high, moderate, low, or very low.

## 3 Discussion

In this article, we present a protocol for systematic review of using moxibustion treatment to treat FC, which is becoming a major public health problem. Current treatments for chronic constipation remain unsatisfactory in therapeutic effect and produce uncomfortable side effects.^[15,37–39]^ Moxibustion, as a traditional method of complementary and alternative medicine methods, has gained increased popularity in patients because of its simple operation, low cost, long curative effect, and no obvious side effects. However, there are no systematic reviews related to moxibustion for FC. The strength of this review lies in that the results will give an overview of current evidence on moxibustion treatment for FC patients. The limitation of this review may be: although we will collect the relevant literature without language restrictions through an extensive and unbiased search of various databases, we cannot be certain that our search will include all relevant RCTs. Additionally, we may have difficulty in retrieving raw data from published sources. The publications or reports we select to search is another possible major cause of bias.

This protocol described here is for the first systematic review and meta-analysis on the efficacy and safety of moxibustion for FC patients. We anticipate to provide an evidence-based basis for moxibustion treatment of FC, and provide useful information to practitioners, policymakers, and patients.

## Author contributions

**Conceptualization**: Ying Chen, Mingmin Xu, Ying Li.

**Data curation**: Ying Chen, Mingmin Xu, Ying Li.

**Formal analysis**: Ying Chen, Mingmin Xu.

**Funding acquisition**: Ying Li.

**Investigation**: Ying Chen, Xiumei Feng.

**Methodology**: Ying Chen, Mingmin Xu, Ying Li.

**Project administration**: Ying Chen, Ying Li.

**Supervision**: Ying Chen, Mingmin Xu, Ying Li.

**Validation**: Lu Wang, Tinghui Hou, Xiumei Feng.

**Visualization**: Ying Chen, Mingmin Xu, Tinghui Hou, Lu Wang, Xiumei Feng, Ying Li.

**Writing** – **original draft**: Ying Chen.

**Writing** – **review & editing**: Mingmin Xu, Ying Li.
